# Vascular Signalling

**DOI:** 10.3390/cells12162038

**Published:** 2023-08-10

**Authors:** Silvia Dragoni, Patric Turowski

**Affiliations:** Institute of Ophthalmology, University College London, 11-43 Bath Street, London EC1V 9EL, UK

In all vertebrates, closed blood and open lymph circulatory systems are essential for the delivery of nutrients and oxygen to tissues, waste clearance, and immune function. Endothelial cells (ECs) line the vessel walls and regulate the passage of molecules and cells to and from the underlying tissue. Mural cells, such as vascular smooth muscle cells and pericytes, imprint vital characteristics onto the vasculature. Significant physical differences are seen between arteries and veins, whereas functional and morphological differences are illustrated by filtrating microvessels in the kidney and the highly impermeable blood–brain barrier (BBB). The vasculature is highly dynamic, as it has complex cellular programs that regulate typification, new blood vessel formation through vasculogenesis or angiogenesis, lumen formation, and EC migration in mature vessels. Recent data also demonstrate the intimate relationships between dynamic vascular functions and metabolic demands and availability. Importantly, regarding human disease, vascular abnormalities underly or accompany the majority of cardiovascular diseases, such as arteriosclerosis, as well as a variety of a priori non-vascular diseases, such as cancer, inflammation, neurodegeneration, or metabolic disease.

Central to the homeostatic and pathological mechanisms of the vasculature are highly specific intra- and inter-cellular signalling pathways, as well as genetic programmes, which are exquisitely responsive to growth factors, cytokines, nucleic acids, and lipids. This Special Issue highlights several important recent developments.

Since its discovery in the 1980s, Vascular Endothelial Growth Factor (VEGF) has been vital to our understanding of the regulation of vascular function. While its basic functions and receptors have been widely studied, many insights remain to be discovered. To exert its functions, VEGFA binds to its cognate receptors, namely VEGFR1 (FLT1) and VEGFR2 (KDR), and initiates the formation of a multiprotein complex that includes co-receptors such as neuropilin-1 (NRP1) and heparan sulfate proteoglycans (HSPGs) [1].

Here, Dallinga et al. investigated the role played by HSPGs and NRP2 in the different steps of VEGFA-induced angiogenesis [2]. Using VEGFA isoforms with different HSPG-binding properties, they find that HSPG engagement appears not to be required for sprouting angiogenesis, tip cell formation, or proliferation of non-tip cells in vitro. In the same system, NRP2 regulates VEGFA–VEGFR2-induced sprout initiation, but it does not regulate tip cell induction. Finally, very low density lipoprotein (VLDL), the endothelial uptake of which is regulated by HSPGs, leads to more sprouts, but it does not lead to increased tip cell formation, suggesting that a connection exists between angiogenesis and metabolism (see also below). Endomucin (EMCN), which is a type I integral membrane glycoprotein of the glycocalyx, interacts with VEGFR2 and regulates its internalisation and downstream signalling response [3]. In this Special Issue, D’Amore et al. describe follow-up studies that characterise molecular determinants of EMCN–VEGFR2 interaction. Specifically, they define a minimal EMCN extracellular domain that is sufficient for VEGFR2-mediated endothelial function, before discussing the importance of two N-glycosylation sites for internalisation and angiogenic activity [4]. Context-specific auto- or trans-phosphorylation of tyrosine residues in the cytoplasmic tail of VEGFR2 initiates various intracellular signalling programmes associated with VEGFA stimulation of ECs [5,6]. In this process, Src homology-2 (SH2)-containing adaptor proteins determine the specificity and selectivity of the response. SH2 domain adapter protein B (SHB) plays connects VEGFR2 stimulation to the activation of focal adhesion kinase (FAK) in angiogenesis and vascular permeability. Here, Welsh and co-workers conducted internal reflection fluorescence microscopy in cultured cells and show that VEGFA stimulation rapidly triggers co-localisation of VEGFR2 with SHB, as well as SHB with FAK [7]. Significantly, FAK co-localisation with VEGFR2 is disturbed in the absence of VEGFR2 phosphorylatable Y1175 or SHB, proving the role of SHB in VEGFR2-mediated FAK activation.

Angiogenesis is not only regulated by VEGFA and its receptors. Pathological release of ATP, including during hypoxia and inflammation, triggers angiogenic expansion of vasa vasorum ECs (VVECs) [8]. In this context, Strassheim et al. investigated ATP-inducible transcription factors that are regulated by the PI3K-Akt-mTOR pathway to examine their role in VVEC angiogenesis [9]. Experiments performed in cultured VVECs identify c-Jun, Foxo3a, and c-Myc as critical downstream regulators of an ATP-activated PI3K-Akt-mTOR pathway. The TGFβ/BMP pathway also modulates angiogenic responses [10]. In this Special Issue, Hiepen, Mendez, and Knaus review emerging evidence of the way in which TGFβ/BMP pathway activation crosstalks with mechanical stimulation in tipping ECs to enable either a homeostatic or an angiogenic response [11]. Importantly, the authors devote considerable focus to the different subcellular localisation of TGFβ/BMP triggers required to enable a concerted endothelial response.

Amongst mural cells, pericytes have long been known to be indispensable supporters and regulators of vascular function, including sprouting and blood vessel formation. More recently, the cytological complexity of pericytes has been recognised, and via this process, the intricate regulatory signalling crosstalk between mural cells and the endothelium occurs, which regulates barrier function, blood flow via lumen diameter, and vessel dropout and repair during ischemic disease. Schmitt et al. investigated whether recognised angiogenic functions of Casein Kinase 2 were also attributable to pericytes [12], finding that in cultured pericytes, CK2 regulates Nerve/Glial Antigen (NG)2-mediated transcription. Furthermore, they provide evidence that genetic and pharmacological inhibition of the pericyte CK2-NG2 axes has profound effects on all parameters of in vitro angiogenesis.

Pericyte also play a major role in establishing barrier function in ECs. In particular, in blood vessels that line the CNS and the retina, they regulate the suppression of endothelial transcytotic pathways [13]. The review by Battaglia et al. discusses the process of transcytosis in brain ECs. It focuses on the intracellular trafficking mediated via tubulation and the role of two actors that have not been properly studied in this context, namely BAR protein and the glycocalyx [14]. Notably, caveolae and fenestrae are absent from blood-brain and blood-retinal barrier ECs. Very little is known about the regulated biogenesis of fenestrae. Ju et al. investigated the cytoskeletal complex that regulates the formation of endothelial fenestrae [15]. Using the actin-depolymerising agent latrunculin A (LtA) to induce fenestrae formation, the authors demonstrate that the actin-binding proteins moesin and annexin II are positive and negative regulators of fenestra formation, respectively. Moesin promotes the assembly of a fodrin cytoskeleton complex linked to the fenestral pore by the proteins PV-1 and Na,K-ATPase.

Another research paper included in this Special Issue investigated the effect of the amyloid-β precursor protein (APP) on ECs and their barrier functions [16]. Ristori et al. observe that APP is necessary for EC proliferation, migration, and adhesion and mediated the EC response to VEGF. Loss of APP alters focal adhesion stability and cell–cell junctions’ expression, thus having a vasoprotective role that should be further investigated.

Besides molecules, cells can also cross the endothelial barrier. Transendothelial migration (TEM) of leucocytes occurs during inflammation and the homeostatic immune surveillance of tissues. During the cascading adhesion of leucocytes to the endothelium, the interaction between leucocyte integrins and the endothelial adhesion molecule ICAM-1 must occur to mediate firm adhesion before diapedesis can be initiated [17]. Morsing et al. demonstrate that during neutrophil transmigration, the disintegrin and metalloproteinase ADAM10 cleaves the extracellular domain of ICAM-1 from the endothelial surface, releasing neutrophils from the endothelium and, thus, facilitating subsequent diapedesis [18]. In a study conducted by our own group, we identified a specific role for the protease activated receptor 1 (PAR1), which is a GPCR, in support of lymphocyte TEM across BBB ECs. PAR1 stimulation leads to the activation of eNOS via AMPK, with consequent modulation of the adherens junction protein VE-cadherin on Y731, thus allowing it to integrate into a previously established TEM signalling pathway mediated by ICAM-1 [19].

Research into the development and pathophysiology of the vascular bed in the retina has accelerated considerably over the past 10–15 years, and the reasons for this shift are primarily related to the intrinsic properties of the retina that make it a suitable research model. Firstly, due to its similarity to the BBB vasculature yet greater accessibility, the retinal vasculature is increasingly being used to study the biology of blood–neural barriers [20]. Secondly, vascular development in the mouse retina occurs post-natally and, thus, has allowed unprecedented genetic and dynamic studies of physiological angiogenesis [21]. Lastly, although anti-VEGFs were originally developed to block tumour angiogenesis, their therapeutic success in treating various retinopathies with dysfunctional vascular aetiologies has been significant [22]. Consequently, many pioneering observations regarding vascular cell biology are increasingly being made in retinal vascular models. Rossato et al. investigated the role of fibrotic changes and endothelial-to-mesenchymal transition (EndoMT) in influencing the therapeutic effects of anti-VEGFs in age-related macular degeneration (AMD). They find that EndoMT induction in primary human retinal ECs correlates with responsiveness to VEGFA [23]. Since fibrotic changes and EndoMT are involved in the progression of choroidal neovascularisation and exacerbated by VEGFR2 inhibition, this result could at least in part explain the reduced efficacy of anti-VEGFA treatment over time.

Additionally, a number of reviews included in this Special Issue focus on important aspects of ocular cell biology and signalling. Microglia are resident immune cells that are distributed in the plexiform layers, ganglion cell layer, and nerve fiber layer of the adult retina. They exert their surveillance function by surrounding the environment with their motile processes. Alves et al. discussed the current knowledge regarding microglial interaction with the retinal vascular system under physiological and pathological conditions [24]. They first focus on the role of microglial cells in the formation and maintenance of the retinal vasculature system, before studying the molecular signalling mechanisms through which microglial cells contribute to the alterations in retinal and choroidal vasculatures and the neovascularisation in AMD.

Eskandarpour et al. review the mechanisms behind non-infectious posterior uveitis (EAU) and highlight the hypothesis of a possible involvement of Leukotrienes (LT) in the genesis of the disease [25]. Recent findings have demonstrated that targeting the high-affinity LTB4 receptor (BLT1) pathway in EAU prevents tissue damage and retinal complications, suggesting that the LTB4 pathway is a promising new therapeutic target for treating intraocular inflammatory diseases. By including preliminary data, the authors hypothesise that VEGFA expression and function in retinal inflammation during EAU is LTB4-dependent.

Lastly, Pouw et al. provide a comprehensive review of the role of the ECM in the eye in influencing health and disease progression [26]. ECM layers are found throughout the eye and play key roles in the cornea, the vitreous, the retinal vasculature and the separation of the neuroretina from the choroidal circulation. Changes to the ECM can have dramatic effects on the homeostasis of ocular function and the pathogenesis of most ocular diseases.

While EC energy generation during angiogenesis is dominated by glycolysis [27], mitochondrial respiration plays a role in cellular stress [28] and specialised vascular beds such as the BBB [29]. Different energy and biomass production requirements play essential roles in this metabolic diversification [30]. Not only are endothelial processes metabolically controlled, but ECs also regulate the metabolic functions of underlying parenchyma [31]; ECs have increasingly been recognised as key contributors to the pathogenesis of metabolic disorders [32]. In this context, Bosseboeuf and Raimondi provide a comprehensive overview on endothelial functions, focusing on recent advances in endothelial signalling and metabolism in physiological conditions and vascular diseases [33]. The highlights of their paper are the sections describing signalling pathways promoted by the transmembrane protein Neuropilin-1 (NRP1) and its recently discovered role in regulating mitochondria and iron homeostasis in both health and pathologies, such as atherosclerosis and neurodegenerative diseases.

The study conducted by Chatterjee et al. focuses on hyperglycaemic ECs [34]. Here, the authors investigated the way in which deficiency of nucleoside diphosphate kinase B (NDPK-B), which affects ECs in a similar way to hyperglycaemia, alters glucose metabolism towards the hexosamine biosynthesis pathway (HBP). Their data indicates that NDPK-B plays a critical role in endothelial damage via the modulation HBP. Moving towards metabolic disease, the review by Paavonsalo et al. summarizes the recent literature on the topic of capillary rarefaction observed in obesity and metabolic diseases [35]. Interestingly, vascular dysfunction and loss of capillaries associated with metabolic diseases is highly organ specific, and this review further supports a key theme of current vascular research, namely that vascular beds are highly diversified and adapted to the tissue that they ultimately serve, thus increasing the complexity and granularity of the way in which we study vascular physiology and biology [36].

Ischemic damage leads to the underlying vasculature of tissues requiring repair and regeneration. The potential of endothelial progenitor cells (EPCs) or endothelial colony-forming cells (ECFCs) to regenerate vasculature and tissues in the context of various ischemic pathologies has been recognised for some time; however, clinical trials in humans using such cells have yielded mixed results, indicating that a greater molecular understanding of endothelial forming cells is required [37]. Two reviews in this Special Issue assemble and critically discuss current knowledge of important aspects in this field. Perrotta et al. focus on EPC/ECFC biology in general, albeit putting a particular focus on the dominant pathways and epigenetic modifications that may influence the therapeutic potential for regeneration following cardiovascular ischemic disease [38]. They provide a very structured overview that takes into account controversies that may have arisen due to poorly aligned nomenclature and protocols. On the other hand, Negri et al. focus on the structure and functions of the TRPV1 channel in ECs and EPC/ECFCs [39]. Specifically, they discuss the role of TRPV1 in angiogenesis, highlighting a novel approach to optical stimulation that make it easier to target this receptor in the treatment of ischemic diseases.

Collectively, the articles published in this Special Issue reflect the importance of vascular signalling to our understanding of key biological and physiological principles.
